# The looming threat of invasive fungal infections in heart transplant recipients: Will it be the last of us

**DOI:** 10.1016/j.jhlto.2026.100482

**Published:** 2026-01-13

**Authors:** Adam Lowe, S. Travis King, Jonathan Hand

**Affiliations:** a University of Arizona College of Medicine-Phoenix, Phoenix, AZ; b Ochsner Health, University of Queensland School of Medicine, Ochsner Clinical School, New Orleans, LA

**Keywords:** Fungal infections, Antifungal agents, Antifungal resistance, Mold, Transplant

## Abstract

Patients with mechanical circulatory support (MCS) devices and those who undergo heart transplants are at risk for invasive fungal infections (IFI). Though *Candida* and *Aspergillus spp.* account for most infections, the evolving epidemiology of resistant-*Candida spp*. such as *Candida auris*, endemic mycoses, and other molds impacted by climate change, threaten MCS and transplant patients. Fungal diagnostics are overall limited, and current, widely used antifungal agents are suboptimal with significant toxicities. However, innovative diagnostic strategies coupled with novel antifungal agents for prevention and treatment of invasive fungal disease encourage optimism for MCS and transplant patients and clinicians.

## Background

Tens of thousands of patients receive mechanical circulatory support (MCS) devices annually, including ventricular assist devices (VAD), Impella devices, and extracorporeal membrane oxygenator cannulation, while over 4,000 additional patients undergo heart transplants each year.^1^, ^2^ Heart transplant recipients taking lifelong immunosuppressive therapy are at increased risk for invasive fungal disease with an estimated cumulative incidence of up to 10%.^3^, ^4^

Fungi, including both yeasts and molds, contribute to approximately 6.5 million severe fungal infection cases annually.^5^ While most people exposed to fungi do not develop serious health issues, those who are critically ill or immunocompromised are particularly vulnerable, experiencing over two million cases of invasive disease each year, which can be associated with significant morbidity and mortality with substantial impacts on global health.^3^, ^5^

This review addresses the escalating issue of invasive fungal infections in heart transplant recipients and patients requiring MCS, highlighting current epidemiology, risk factors, outcomes, as well as treatment and management challenges and potential future directions. Increased awareness and updated data are crucial to mitigate these infections and enhance patient outcomes.

### Epidemiology, risk factors, and outcomes in heart transplant and mechanical circulatory suppor

The increased utilization of MCS, particularly in patients transitioning or successfully undergoing heart transplantation, presents a significant challenge with regard to fungal infections. Surveillance data indicate that the incidence of invasive fungal infections post-heart transplant can reach between 3% and 11%, with some single-center cohort reporting cumulative incidence up to 10.7%.^3^, ^6^, ^7^, ^8^ Similar incidence has been described in transplant programs in Europe and Latin America, underscoring that IFIs are a global complication of heart transplantation.^7^ Patients supported by VADs have a notably higher infection rate of up to 23%.^3^, ^6^, ^7^, ^8^ Additionally, studies from the Extracorporeal Life Support Organization Registry report approximately 10.8% of adults on extracorporeal membrane oxygenator exhibited fungal colonization or infection, illustrating the critical need for vigilant monitoring and proactive management of fungal infections in this patient population.^9^

The highest incidence of IFI in heart transplant recipients occurs within the first 3 months post-transplantation.^4^, ^10^, ^11^ The most prevalent organisms include *Candida* (53%), *Aspergillus* (19%), and *Cryptococcus spp.* (8%).^6^, ^8^, ^11^ While less common, endemic fungi such as Histoplasmosis, commonly found in the Ohio and Mississippi river valleys, Blastomycosis, found in central and southeastern United States, and Coccidioidomycosis, found in the southwestern United States, may also lead to post transplant infection.^12^ Infections caused by Mucorales, Scedosporium/Lomentospora spp., Fusarium spp., and dematiaceous fungi are more commonly observed in lung transplant recipients compared to heart transplant recipients, where such infections are rare.^13^, ^14^

While degree of immunosuppression, post-transplant renal replacement therapy, and the use of anti-thymocyte globulin are key risk factors associated with IFI, a number of others exist unique to certain organisms.^3^, ^4^, ^8^, ^15^, ^16^ Most notable risk factors in *candida* infections include prolonged antibiotics, temporary MCS, and post-transplant renal replacement therapy.^3^, ^4^ In cases involving cryptococcus, history of allograft rejection and environmental exposure to bird droppings are common factors, and in the case of aspergillosis, concurrent CMV infection and pre-transplant colonization increase risk.^4^, ^8^, ^15^, ^16^ For MCS patients, factors such as invasive respiratory support, recent influenza infections, sepsis, and total parenteral nutrition in those with VADs further elevate the risk of invasive fungal infections.^6^, ^9^

Fungal infections pose a serious threat to both adult and pediatric patients following cardiac transplantation, with mortality rates notably high. In a historical (1985-1997) Italian, adult cohort mortality in patients with aspergillosis and candidiasis were 29.4% and 33.3%, respectively.^17^ More recently a population-based cohort study of solid organ transplant recipients in Ontario, mostly with aspergillosis and candidiasis, reported a 1-year all-cause mortality of 34.3%.^18^ Data from a multi-institutional registry study evaluating IFI in pediatric heart transplant recipients found 49% of patients with infection died within 6 months.^19^ In patients on MCS, particularly VADs, mortality rates range from 35.7% to 91%.^20^ This underscores the urgent need for effective prevention and management strategies in these vulnerable populations.

### Current management challenges

Management of invasive fungal infections has historically been accomplished using a small assortment of antifungal agents, most notably the systemic 14-a demethylase inhibitors (azoles), glucan synthase inhibitors (echinocandins), and amphotericin B. These medications have accounted for hundreds of thousands of treatment successes - and failures - but come at the expense of unfavorable pharmacokinetics and adverse events/toxicities.

The azole antifungal class possesses a broad spectrum of activity that includes *Candida spp.* (fluconazole - excluding Candida krusei), Aspergillus spp. (voriconazole, posaconazole, and isavuconazole), Mucorales (posaconazole and isavuconazole), Fusarium spp. (voriconazole, posaconazole, and isavuconazole), and Scedosporium/Lomentospora spp. (voriconazole). These medications also present significant problems in MCS and heart/lung transplant patients given the risk of significant drug interactions with immunosuppressive medications (calcineurin inhibitors) and the risk of prolonging the QTc interval. It is important to note that isavuconazole shortens the QTc. Four major enzymes or transporters are involved in azole-associated metabolism and inhibition: 2C9, 2C19, 3A4, and P-glycoprotein.^21^ The degree of inhibition differs within the class. Table 1 lists the drug-enzyme interactions amongst the triazole antifungals. In this table, “inhibitor” refers to drugs that reduce the activity of the listed CYP enzyme, thereby decreasing metabolism of co-administered substrates. “Inhibitor & substrate” refers to drugs that are both metabolized by and inhibit the same CYP enzyme. Voriconazole stands out in the class given its highly variable pharmacokinetics. In addition to being a substrate/inhibitor of 2C9/2C19, it is also subject to pharmacogenomic variability in its metabolism.^22^ This results in a wide range of serum concentrations that may result in clinical toxicity or lack of efficacy. These genomic variations, as well as poor absorption for other class members (itraconazole, posaconazole suspension) has resulted in the use of therapeutic drug monitoring (TDM) for these agents to maximize efficacy and/or minimize toxicity. Routine TDM is not generally recommended for fluconazole and isavuconazole.^23^ Taken together, these properties make a case for the need for newer classes of antifungals to expand the repertoire.

**Table 1:** Drug-Enzyme Interactions With Triazole Antifungals

	Antifungal	Fluconazole	Voriconazole	Posaconazole	Itraconazole	Isavuconazole
Enzyme						
2C9		Inhibitor	Inhibitor substrate	--	Inhibitor	
2C19		Inhibitor	Inhibitor substrate	--	--	
3A4		Inhibitor	Inhibitor substrate	Inhibitor	Inhibitor substrate	Inhibitor substrate
P-GP*				Inhibitor substrate	Inhibitor	

*P-glycoprotein.^21^, ^24^

The echinocandin antifungals have a more limited role in the management of invasive fungal infections. Clinically, their use is limited to first-line management of invasive candidiasis/candidemia and salvage or combination treatment of Aspergillus spp.^25^ While clinical data assessing echinocandins against Mucorales is largely restricted to case reports, in vitro and in vivo (murine) analyses have shown synergy with other Mucorale-active antifungals.^26^, ^27^, ^28^ Given that their therapeutic target is specific to fungi, they lend themselves to being largely devoid of major toxicities and drug interactions. The class shares pharmacokinetic similarities of poor oral bioavailability, non-renal metabolism, and an inability to penetrate sanctuary tissues (vitreous, central nervous system (CNS)).^25^ TDM is not needed for this class.

Amphotericin B (AmB) is a polyene antifungal that targets ergosterol in the cell membrane of fungi. It has a sweeping spectrum of activity that includes those mentioned for the azoles and echinocandins and has been the mainstay of treatment for invasive yeast and mold infections for decades.^25^ Of note, it lacks meaningful activity in *C lusitaniae*, *A terreus*, and Lomentospora spp.^25^, ^29^ While amphotericin does not generally pose concerns for drug-drug interactions, it is associated with significant adverse events that limit its use, including an infusion-related syndrome, electrolyte abnormalities, and kidney injury.^25^ Fluid boluses, specifically saline loading, are given before and after the infusion to minimize the insult on the nephrons. Newer formulations of AmB have utilized lipid binders, which have shown promise in minimizing adverse events and facilitating higher dosing regimens.^25^

### New and emerging fungal threats

The Centers for Disease Control and Prevention and World Health Organization have identified multiple fungi, including *Candida auris,* drug-resistance *Candida spp*., and azole-resistant Aspergillus spp., as critically important pathogens posing significant global threats.^30^, ^31^ These emerging threats may disproportionately impact MCS and heart transplant recipients.^3^, ^4^, ^32^, ^33^, ^34^, ^35^, ^36^

*Candida auris* has rapidly spread in healthcare settings worldwide and has been responsible for up to 40% of candidemia cases in certain countries.^33^, ^35^, ^36^ Organ transplantation has been identified as a risk factor due to prolonged hospital stays, exposure to broad-spectrum antibiotics, and the presence of indwelling catheters.^35^, ^36^ This organism has unique characteristics that facilitate growth in high salt and temperature settings, supporting its environmental persistence. Further, laboratory misidentification of *C auris* is common, and its resistance to multiple classes of antifungals making management and treatment of infections extremely challenging. Though echinocandins are typically recommended for treatment of *C auris* infections, resistance rates are rapidly rising, with 30% of isolates reportedly resistant to AmB.^32^, ^33^, ^35^, ^36^ Similarly, healthcare-associated outbreaks of fluconazole-resistant *C parapsilosis* have been described in immunocompromised patients.^35^

Climate change has impacted the evolving epidemiology of microorganisms across the world and may play a significant role in the rapid, worldwide presence of *C auris*.^37^ Additionally, recent geographic expansion of endemic fungi, such as Histoplasma spp., Blastomyces spp., and Coccidiodes spp., are thought to be influenced by the warming climate.^37^ Given the changing epidemiologic patterns of these fungal organisms, which are associated with significant morbidity and mortality in heart transplant recipients, clinicians may consider having a lower threshold to test if patients present with a clinical syndrome compatible with invasive disease.^38^ Likewise, changing epidemiology may also impact pre-transplant screening in patients who have spent time or reside in Coccidioides-endemic regions.

Furthermore, climate change has enhanced the global risk for natural disasters. Though not completely understood, increases in invasive mold infections after natural disasters have been well documented, and transplant recipients living in susceptible regions of the world are at risk.^37^ Infections may occur after wildfires, tsunamis, floods, and hurricanes by direct inoculation from trauma, inhalation of molds from airborne dispersal, or mold growth inside of damaged homes.

Antifungal resistance is a growing concern in transplant recipients. Though multifactorial, resistance may be impacted by the widespread use of prophylactic and treatment antifungals in transplant populations.^39^ Though universal antifungal prophylaxis for *candida* and invasive aspergillosis after heart transplant is typically not recommended in consensus guidelines, targeted prophylaxis may be considered based on local epidemiology.^32^, ^40^ More recently described antifungal stewardship, often under the umbrella of institutional antibiotic stewardship programs, has emerged as a multifaceted strategy to improve antifungal prescribing and avoid consequences of inappropriate use such as toxicities and antifungal resistance.^41^ Though some countries have well-established antibacterial resistance surveillance systems that may capture antifungal resistance, these programs need optimization and further expansion. Finally, worldwide agricultural fungicide use, specifically azoles, has also been linked to environmental and clinically significant fungal resistant pathogens which may further impact the capability to care for heart transplant recipients with such infections.^41^

Access to medical and recreational inhaled marijuana is expanding across the world, and case reports of associated invasive mold infections have been published.^42^ Though no large, methodologically robust studies have been done to better understand infectious risk, nearly 50% of 225 transplant providers surveyed recalled marijuana-related fungal infections in their transplant recipients.^42^ A recent report found donors with a history of recent marijuana did not portend infectious risks to recipients in the early post-transplant period.^43^ As more patients who become donors or undergo heart transplants have less restrictive access to marijuana, it will be important to further clarify the epidemiology and risk of marijuana-related fungal infections.

### Emerging fungal threats: Donor impacts

Though overall rare, donor-derived fungal infections are associated with significant morbidity and mortality in transplant recipients. In a recent report summarizing 10 years of Disease Transmission Advisory Committee experience, 54 proven or probable fungal infectious transmission were reported, with 6 occurring in heart transplant recipients.^44^ *Candida auris* has been transmitted through lung transplantation, and the optimal management of donors with infection and strategies to screen for colonization have not been described.^45^ Additionally, outbreaks of Fusarium meningitis have occurred. In a recent report, 5 recipients safely received an organ from a donor with this infection without signs of recipient infection after 4 of 5 received voriconazole prophylaxis.^46^ A recent review of donor-derived endemic mycoses found these infections are often disseminated and associated with high mortality.^47^ It is unclear how climate change-generated expansion of fungi, including endemic mycoses, will impact donor-derived infections.

### Emerging fungal diagnostics

Diagnosing IFIs (e.g., candidiasis, aspergillosis) in patients with MCS or after heart transplant is frequently challenging. Cultures (blood, tissue, and fluid) have variable sensitivity (∼50% for invasive candidiasis) and may be frequently sent to reference labs, limiting turnaround times.^48^ Further, obtaining tissue specimens may not be feasible or safe in patients with critical illness and other comorbidities. When *Candida spp*. are present in non-blood cultures, it can be challenging to distinguish colonization from true invasive infection. Though fungal biomarkers such as a serum β-D-glucan or galactomannan assays may be used as adjunctive IFI diagnostics, they exhibit limited and variable sensitivity and specificity.^34^, ^48^

Fungal biomarkers, antibody tests, and myriad rapid, molecular identification methods for detecting *Candida spp*. from blood culture are commercially available (e.g., Biofire FilmArray BCID, GenMark ePlex BCID-FP, MALDI-TOF MS, Accelerate Pheno System, T2 Biosystems T2Candida Panel).^49^, ^50^ Nucleic acid amplification tests for Aspergillus spp. and Mucorales from bronchoalveolar lavage specimens are commercially available and widely used.

Though large, well-designed studies are limited, experiences using metagenomic next-generation sequencing (e.g., Karius) to diagnose IFIs have been published.^50^ Freeman Weiss et al have also recently summarized novel testing for fungal infections including fungal point-of-care testing using lateral flow assays, which may provide rapid and affordable opportunities to expand access to fungal diagnostics.^49^ Finally, to improve understanding of antifungal resistance impacting treatment and our understanding of epidemiology, antifungal susceptibility testing of *Candida spp*. and molds must improve and expand, as commercially available platforms for testing molds are limited.^50^

Positron emission tomography-computed tomography with 18F-fluorodeoxyglucose can be highly sensitive for diagnosis and staging of bacterial left ventricular assist device infections and support management of IFI in immunocompromised patients.^51^, ^52^ Non-specific fluorodeoxyglucose uptake may limit interpretation of IFI diagnoses, and more robust, well-designed studies are needed to better understand and define optimal use of this imaging modality.

### Novel prevention and therapeutic strategies

In addition to the medications mentioned above, several novel agents have been recently approved or are being studied in late-stage trials for invasive yeast and mold infections. These agents display a broad and differing spectrum of activity that complement the current, limited options for patients with IFI. A summary of these agents can be found in Table 2.

**Table 2:** Novel Agents Recently Approved or Being Studied in Late-Stage Trials for Invasive Yeast and Mold Infections

Generic name (Brand Name)	Mechanism of action	Spectrum of activity	Recommended dosing	Drug-drug interactions	Interactions with anticoagulation	Adverse events	References
Ibrexafungerp (Brexafemme)	Triterpenoid inhibitor of 1,3-β-D-glucan synthase	*Candida* spp. (including azole- and echinocandin-resistant strains and *C auris*) Aspergillus spp.	Vulvovaginal Candidiasis: 300 mg orally twice daily for 1 day.	Substrate of CYP3A4. Reduce dose to 150 mg twice daily when given with strong CYP3A4 inhibitors. Use not recommended with strong CYP3A4 inducers.	No specific interactions with heparins, warfarin, DOACs. Routine anticoagulation can generally be continued with usual clinical monitoring	Nausea, diarrhea, abdominal pain Contraindicated in pregnancy	^53^
Rezafungin (Rezzayo)	Echinocandin inhibitor of 1,3-β-D-glucan synthase	*Candida spp*, (including azole- and echinocandin-resistant strains and *C auris) Aspergillus spp.*	Invasive Candidiasis/Candidemia: 400 mg IV as a loading dose on Day 1, followed by 200 mg IV once weekly	None recognized	No specific interactions	Infusion-related reactions, hypokalemia, photosensitivity, elevated liver function tests	53, 54
Olorofim (investigational)	Inhibitor of dihydroorotate dehydrogenase	*Aspergillus spp. Scedosporium, spp. Lomentospora prolificans.* Not active against *Candida* or *Mucorales*.	150 mg IV twice on day 1 followed by 90 mg twice a day	Substrate of CYP3A4. Caution when used with CYP3A4 inhibitors/inducers	No specific clinically important interactions with anticoagulant reported	Nausea, vomiting, diarrhea, elevated liver function tests	53, 54, 55
Fosmanogepix (investigational)	Inhibitor of fungal Gwt1	*Candida* spp. (except *C krusei*), *Aspergillus spp., Fusarium spp., Cryptococcus spp*., and *Scedosporium spp.*	1,000 mg IV twice daily on Day 1, followed by 600 mg IV once daily	None recognized	No specific interactions reported	Nausea, headache, flushing	53, 54, 56

Fosmanogepix, the prodrug of manogepix, represents a new class of antifungals that inhibit the inositol acyltransferase Gwt1, resulting in faulty glycosylphospatidylinositol membrane incorporation and mannoprotein anchoring to the cell wall.^53^ In vitro susceptibility studies have shown fosmanogepix to have broad activity against *Candid*a spp with the exception of *C krusei*.^57^ Additionally, some cryptococcal, aspergillus, scedosporium/lomentospora, and fusarium isolates have demonstrated susceptibility. Notably, robust activity against agents of the Mucorales order is variable to lacking. Pre-clinical studies have shown fosmanogepix to have favorable tissue penetration, including the central nervous system and the eye.^58^ A phase 2 open-label study demonstrated promising outcomes in the treatment of invasive *candid*iasis with no discontinuations due to adverse events. A phase 3 trial for treatment of invasive candidiasis is ongoing.^59^ Fosmanogepix is being developed as both intravenous and oral preparations.

Ibrexafungerp is a first-in-class triterpinoid antifungal whose mechanism resembles that of the enchinocandins. Ibrexafungerp binds to 1,3-beta-D-glucan synthase but at a site that differs from the echinocandins.^53^ Similar to the echinocandins, it has broad activity against Candida spp. including *C auris*. Ibraxafungerp is also fungistatic against Aspergillus spp. Ibrexafungerp’s alternate binding site on glucan synthase allows it to retain activity against echinocandin-resistant organisms (FKS1/FKS2). It does not have activity against Fusarium spp. or the Mucorales. Pre-clinical models have shown ibrexafungerp to have excellent peripheral tissue distribution, but penetration into the CNS is poor. It is a substrate of CYP3A4 and is known to inhibit CYP2C8 and CYP 3A4; however, dose alterations are generally not needed with the exception of strong CYP3A4 inducers, which are contraindicated due to reductions in ibrexafungerp exposure.^53^ This antifungal is approved by the Food and Drug Administration for the treatment of vulvovaginal candidiasis, and trials are ongoing for the treatment of invasive candidiasis. Ibrexafungerp is commercially available as an oral formulation.

Olorofim represents yet another novel-class antifungal agent under development for invasive disease. Olorofim inhibits the enzyme dihydroorotate dehydrogenase, resulting in a reduction in pyrimidine synthesis.^53^ Importantly, olorofim does not appear to have any significant cross-reactivity with human variants of this enzyme. Olorofim does not possess meaningful activity against *Candida spp*. or the Mucorales; however, it is broadly active against Aspergillus spp., dimorphic fungi, and dematiaceous molds. This activity extends to isolates resistant to currently available antifungal agents. It has poor oral bioavailability but has shown good tissue penetration, including brain tissue.^60^ Olorofim is metabolized by CYP3A4; caution should be taken when given with strong CYP 3A4 inducers and/or inhibitors. A recent phase 2 study evaluating olorofim in the treatment of invasive fungal infections in patients lacking options reported an approximate 40%-45% response rate.^54^, ^55^ The predominant molds enrolled were Aspergillus and Scedosporium/Lomentospora.^55^ Olorofim is being developed as an intravenous formulation.

Rezafungin represents the newest addition to the echinocandin class of antifungals and shares a mechanism of action, inhibition of 1,3-beta-D-glucan synthase, with currently available agents.^54^ Rezafungin differs from current agents in a pharmacokinetic manner. Chemically similar to anidulafungin with the addition of a choline aminal ether, rezafungin maintains the activity of other echinocandins but exhibits a half-life of more than 80 hours.^53^ This allows for weekly dosing intervals. Similar to other echinocandins, rezafungin does not affect the CYP systems in any appreciable manner. Additionally, it demonstrates similar, sufficient peripheral tissue penetration and poor penetration of the CNS, vitreous, and urine.^53^, ^61^ Rezafungin’s activity is analogous to other echinocandins in that it is active against a broad population of *Candida spp*., including *C auris*, as well as, Aspergillus spp.^54^ Interestingly, rezafungin has shown activity against pneumocystis in animal models. Rezfungin is commercially available for the treatment of invasive candidiasis and is currently being studied as a prophylaxis option for *Candida spp., Aspergillus spp.,* and pneumocystis in allogeneic stem cell transplant patients.^54^ It is available as an intravenous formulation.

Vaccines to prevent invasive fungal infections (e.g., candidiasis, coccidioidomycosis, cryptococcosis, histoplasmosis, and aspergillosis) using traditional vaccine technology and more novel approaches are currently being studied with some moving into human clinical trials.^62^

## Conclusion

As the overall number of patients undergoing MCS implantation and heart transplantation increases, the post-transplant lifespan extends, the climate changes, and the epidemiology of fungal infection evolves, more research is needed to improve diagnostic testing and surveillance programs. Further, given the rise of antifungal resistance, the creation of novel prevention and treatment interventions is critical for the care of MCS and heart transplant recipients.

## Conflicts of Interest statement

S. Travis King reports a relationship with Melinta Therapeutics LLC that includes: speaking and lecture fees. S. Travis King reports a relationship with Scynexis Inc. that includes: funding grants. Jonathan Hand reports a relationship with Pfizer that includes: consulting or advisory and funding grants. Jonathan Hand reports a relationship with AstraZeneca that includes: consulting or advisory and funding grants. Jonathan Hand reports a relationship with Innoviva Specialty Therapeutics Inc. that includes: consulting or advisory and funding grants. Jonathan Hand reports a relationship with Ferring Pharmaceuticals Inc. that includes: funding grants. Jonathan Hand reports a relationship with Scynexis Inc. that includes: funding grants. If there are other authors, they declare that they have no known competing financial interests or personal relationships that could have appeared to influence the work reported in this paper.
